# Chronic Traumatic Encephalopathy in a Routine Neuropathology Service in Australia

**DOI:** 10.1093/jnen/nlac071

**Published:** 2022-08-10

**Authors:** Catherine M Suter, Andrew J Affleck, Maggie Lee, Danielle Davies, Arran L Burns, Joanne Sy, Bernard I’Ons, Michael E Buckland

**Affiliations:** From the Department of Neuropathology, Royal Prince Alfred Hospital, Camperdown, NSW, Australia; School of Medical Sciences, University of Sydney, Camperdown, NSW, Australia; From the Department of Neuropathology, Royal Prince Alfred Hospital, Camperdown, NSW, Australia; From the Department of Neuropathology, Royal Prince Alfred Hospital, Camperdown, NSW, Australia; From the Department of Neuropathology, Royal Prince Alfred Hospital, Camperdown, NSW, Australia; From the Department of Neuropathology, Royal Prince Alfred Hospital, Camperdown, NSW, Australia; Sydney Medical School, University of Sydney, Camperdown, NSW, Australia; From the Department of Neuropathology, Royal Prince Alfred Hospital, Camperdown, NSW, Australia; From the Department of Neuropathology, Royal Prince Alfred Hospital, Camperdown, NSW, Australia; Forensic and Analytical Scientific Services, Lidcombe, NSW, Australia; From the Department of Neuropathology, Royal Prince Alfred Hospital, Camperdown, NSW, Australia; School of Medical Sciences, University of Sydney, Camperdown, NSW, Australia

**Keywords:** Brain autopsy, CTE, Head injury, Neurodegeneration, Neuropathology, p-tau, Traumatic brain injury

## Abstract

Chronic traumatic encephalopathy (CTE) is a neuropathological diagnosis defined by a unique pattern of hyperphosphorylated tau (p-tau) accumulation that begins in neocortical regions of the brain. It is associated with a range of neuropsychological symptoms, but a definitive diagnosis can only be made by postmortem brain examination. In 2018, we instituted CTE screening for all autopsy brains as part of our routine departmental protocol by performing p-tau immunohistochemistry on a restricted set of 3 neocortical blocks (frontal, temporal, and parietal). This strategy allowed us to identify 4 cases of low-stage CTE from 180 consecutive autopsies. Two of the 4 cases had a documented history of brain injury; for the remaining 2 cases, there was a long history of treatment-resistant tonic/clonic epilepsy suggesting that undocumented brain injuries may have occurred. Our experience indicates that 3-block CTE screening is useful in identifying CTE in routine practice. The results of this study further support the association between prior head injuries and CTE and demonstrate that, albeit uncommon, CTE does occur in the general population. Our findings suggest that p-tau screening should be routinely pursued in brain autopsy, particularly where there is a documented or likely history of traumatic brain injury.

## INTRODUCTION

Chronic traumatic encephalopathy (CTE) is a neurodegenerative disease strongly associated with prior exposure to repetitive head impacts (RHI) (1). Initially described in boxers as “punch drunk” syndrome, the disease has now been identified in amateur and professional athletes from a wide variety of contact sports, as well as others exposed to RHI such as military personnel, victims of domestic violence, and people with treatment-resistant epilepsy (2). CTE can currently only be diagnosed postmortem and is characterized by accumulation of hyperphosphorylated tau (p-tau), “in neurons, with or without thorn-shaped astrocytes, at the depth of a cortical sulcus around a small blood vessel, deep in the parenchyma, and not restricted to the subpial and superficial region of the sulcus” (3). The pathology appears to be progressive (4), with a spectrum of disease severity whereby medial temporal lobe structures are only affected in high-stage pathology (3).

In 2018, we identified the first case of CTE in a retired professional rugby league player (5). To the best of our knowledge, this was only the second case of CTE ever described in Australia (6). Following this diagnosis, we decided to incorporate minimal neocortical p-tau immunohistochemistry (IHC) as routine practice in order to evaluate the prevalence of CTE in our regular neuropathology service at Royal Prince Alfred Hospital (RPAH), Sydney, Australia. The RPAH Department of Neuropathology is the only dedicated clinical neuropathology unit in New South Wales, the most populous state in Australia.

In the absence of a clinical history of cognitive decline or dementia, our previous screening protocols incorporated p-tau immunostaining of the hippocampus only, principally to assess for the presence of Alzheimer disease neuropathologic change (ADNC). Given that such medial temporal lobe structures are not involved until high-stage CTE, this approach means that many or most cases of low-stage CTE would not have been identified. We therefore amended our protocols in 2018 based on a prior study that found the examination of 3 neocortical blocks stained for p-tau is sufficient to detect ∼78% of known CTE cases (7). A similar approach was previously used for a “lookback” study to identify CTE in a neurodegenerative disease brain bank (8). We adapted these approaches and subsequently applied our screening protocol to routine blocks of frontal, parietal, and temporal lobes taken at brain examination.

Also, within the RPAH Department of Neuropathology is the Australian Sports Brain Bank (ASBB; brainbank.org.au). The ASBB, launched in March 2018, uses specialist diagnostic neuropathology, coupled with research, to understand CTE and other brain pathology that is associated with traumatic brain injury (TBI) in sport and elsewhere. We have recently published the results of the first 21 brains donated to the ASBB over a 3-year period, from March 2018 to March 2021 (9). These brain donations were almost all prompted by concerns from the donors or their families over cognitive and/or neuropsychiatric issues affecting the donors. We found that all but one donated brain had some form of degenerative pathology and over 50% (12/21) of the donated brains had pathognomonic CTE pathology (9). Here we detail the results of all completed cases of routine CTE screening over 3 years, from March 2018 until March 2021, the identical time period of completed brain donations to the ASBB (9).

## MATERIALS AND METHODS

### Ethics Approval and Consent to Participate

Autopsy referrals for neuropathological examination come from a wide range of sources that include hospital autopsies, coronial cases, state-wide Creutzfeldt-Jakob disease surveillance, and brain banking for research (including the Australian Sports Brain Bank, ASBB). All departmental procedures and reporting are approved by the National Association of Testing Authorities and RPAH. Brains referred for tissue banking were obtained with informed consent (RPAH HREC #19-0010). Inclusion of coronial cases was approved by the NSW State Coroner. This study was approved by the RPAH HREC (#22-0053).

### Clinical Samples

A total of 192 autopsy referrals accrued over the period of March 2018 until March 2021 are included in this report. The source of individual cases is not revealed to preserve confidentiality. The majority of cases (n = 180) are representative of consecutive brain referrals to the department and included 18 cases donated via the RPAH Neuropathology Tissue and Tumour Bank (NTTB) specifically because of a clinical diagnosis of dementia. Some cases were excluded from p-tau screening. These were: (1) those under the age of 18 years, (2) those with confirmed Creutzfeldt-Jakob disease (for occupational health reasons), and (3) those donated to the Multiple Sclerosis Australian Brain Bank, (as this brain bank operates under different ethical approvals). The remainder of cases included in this report are derived from brain donation to the ASBB (n = 12) and all had a diagnosis of CTE, as reported previously (9).

### Neuropathological Examination

Brains were fixed in formalin for 7–14 days prior to macroscopic examination and tissue sampling. All brains underwent comprehensive sampling based on a standardized protocol encompassing multiple neocortical regions, subcortical structures, hippocampi, brainstem, and cerebellum. Additional regions may have been sampled based on the clinical history and lesions identified. All samples were paraffin-embedded and cut at 4 microns.

For neuropathological diagnosis, requisite histochemical stains and IHC were performed as per our standardized clinical protocol, including p-tau IHC in 3 neocortical regions for all cases, as described below. For ASBB cases, more than 3 neocortical blocks underwent p-tau IHC, with a minimum of 16 blocks for each case examined according to ASBB brain examination protocols. For the purposes of this study, only the 3 screening blocks were examined.

### CTE Screening

For all routine cases, p-tau IHC was performed on 3 neocortical blocks: anterior frontal cortex encompassing superior and middle frontal gyri and the anterior watershed zone (block A), peri-Rolandic cortex (block B), and superior and middle temporal gyri at the level of the anterior hippocampus (block D). For ASBB cases and some NTTB dementia cases, more extensive cortical sampling was performed as per our standard procedure for ASBB donors, or when indicated for NTTB donors with neurodegeneration. p-tau IHC was performed using the AT8 monoclonal antibody (Invitrogen, ThermoFisher) at 1:800 dilution on a Leica Bond-Max autostainer. CTE diagnosis and scoring were performed according to current NINDS/NIBIB consensus neuropathological criteria for CTE (3), where CTE lesions are defined as, “phosphorylated Tau aggregates in neurons, with or without thorn-shaped astrocytes, at the depth of a cortical sulcus around a small blood vessel, deep in the parenchyma, and not restricted to the subpial and superficial region of the sulcus.” All diagnostic neuropathology (with the exception of the 3-block p-tau IHC) was performed by 1 of 3 neuropathologists (MEB, JS, BI). For evaluation of p-tau IHC, the 3 blocks for each case were reviewed independently by 2 observers (MEB, AJA). The observers were blinded to patient identity and clinical history. There was no interobserver discordance with CTE diagnoses in either the routine or ASBB cohorts.

### Clinical Information

Clinical histories were derived from the review of supplied clinical notes, typically from the treating clinician in donor and noncoronial cases, and from the coronial case notes for coronial examinations. Evidence for a history of TBI was recorded if the supplied notes indicated that a brain injury or injuries were sustained at least 12 months prior to death. Evidence for a history of substance abuse was recorded if the notes indicated abuse of alcohol, illicit drugs, or prescription medication at any time prior to death.

### Statistical Analyses

Association between neurodegenerative pathology and history of TBI or substance abuse was assessed using chi-squared test with p < 0.05 considered significant. Odds ratios were calculated according to Altman (10).

## RESULTS

### Cohort Demographics

At the time of writing, CTE screening in our routine practice had been completed and reported on a total of 180 brains included in this report. Relevant cohort demographics are shown in Table 1. Evidence of a history of TBI was present in both males and females (17% and 11%, respectively). The source of TBI was varied and included head injuries sustained via: physical assaults (n = 9), falls (n = 6), motor vehicle accidents (n = 4), neurosurgery (n = 4), sports (n = 2), and intimate partner violence (n = 1). On average, 28% of the cohort (38% of males and 15% of females) had a history of significant substance abuse (alcohol and/or prescription and illicit drugs). These figures are increased relative to the rates of substance abuse in the general Australian population (11). We also recorded whether death occurred by suicide because of the high prevalence of suicide we reported previously in our Australian Sports Brain Bank donors (9). Five individuals in the current cohort (2.8%) died by suicide, and this is consistent with standardized suicide rates in Australia (11).

**TABLE 1.** Cohort Demographics

	All Cases
	(n = 180)
Sex, n (%)	
Male	105 (58)
Female	75 (42)
Age at death in years, avg±SD (range)	
All	55 ± 19 (18–101)
Male	53 ± 18 (18–92)
Female	59 ± 20 (18–101)
History of TBI, n (%)	
All	26 (14)
Male	18 (17)
Female	8 (11)
History of substance abuse, n (%)	
All	51 (28)
Male	40 (38)
Female	11 (15)
Death by suicide, n (%)	
All	5 (2.8)
Male	3 (2.9)
Female	2 (2.6)

SD, standard deviation; TBI, traumatic brain injury.

### Neurodegenerative Pathology

The routine neuropathological findings in the cohorts (with respect to neurodegeneration only) are summarized in Table 2. The majority of cases (128/180, 71%) did not exhibit any neurodegenerative pathology. However, within the cohort of 180, there were 18 cases derived from individuals who donated their brains for dementia research; unsurprisingly, all of these exhibited one or more neurodegenerative pathologies but none exhibited CTE. Alzheimer disease was the most common neuropathology in both the unselected clinical cohort and the subcohort referred because of clinical dementia. No individual in the dementia cohort had a history of TBI or substance abuse; this subgroup was significantly older than the general clinical cohort (75 ± 8 vs 53 ± 19 years at death). While a history of substance abuse was common in the general clinical cohort, it was not associated with the presence of neurodegenerative pathology χ^2^ (1, n = 162)=2.433, p = 0.119. However, a history of TBI was positively associated with the presence of neurodegenerative pathology, χ^2^ (1, n = 162)=7.229, p = 0.007. Prior TBI was associated with a 3-fold increased chance of any neurodegenerative diagnosis, odds ratio 3.3 (95% CI 1.3–7.9). There was no difference in age between the subgroups with respect to TBI history. The average age of those with evidence of TBI history was 61 ± 21 years; the average age of those with no evidence of TBI history was 54 ± 18 years, p = 0.12, Student t-test.

**TABLE 2.** Summary of Neurodegenerative Pathology in Our Routine Practice

Neurodegenerative Pathology, n (%)	Unselected Clinical Cohort	Dementia Donors
	(n = 162)	(n = 18)
None	128 (79)	0
Alzheimer disease	16 (9.9)	12 (67)
Aging-related tau astrogliopathy	12 (7.4)	1
Limbic/neocortical Lewy body disease	6 (3.7)	4 (22)
Chronic traumatic encephalopathy	4 (2.5)	0
Frontotemporal lobar degeneration	3 (1.9)	0
Primary age-related tauopathy	2 (1.2)	0
Motor neuron disease	1 (0.6)	1 (5.6)
Limbic-predominant age-related TDP-43 encephalopathy	1 (0.6)	0
Adult-onset leukodystrophy with spheroids and pigmented glia	0	1 (5.6)

### CTE Neuropathology

**FIGURE 1.**
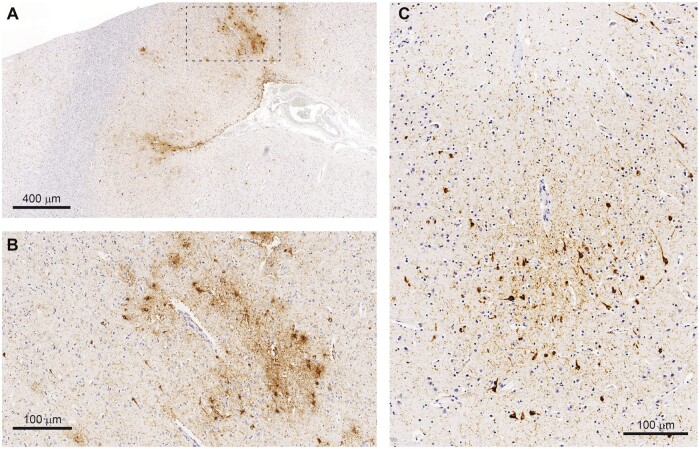
Typical CTE lesions encountered in routine practice. **(A)** Low-power view of CTE lesions from Case 1 showing p-tau deposition in neurons and neurites and astrocytes at the depth of sulci, concentrated around blood vessels. ARTAG is also present in this case in subpial regions. **(B)** High-power view of the perivascular CTE lesion marked by the rectangle in **(A)**. **(C)** High-power view of a CTE lesion from Case 3 demonstrating predominantly neuronal p-tau aggregates around a blood vessel.

Four individuals (1 female, 3 male) in the general clinical cohort were found to harbor pathognomonic CTE lesions in one or more of the 3 blocks screened (Table 3). These lesions carried the hallmark CTE aggregates of p-tau in neurons and neurites at the depth of a cortical sulcus, around a small blood vessel, deep in the parenchyma (Fig. 1). In the unselected general clinical cohort, the prevalence of CTE (2.5%) sat between frontotemporal lobar degeneration (1.9%) and Lewy body disease (3.7%). Three of the 4 CTE cases also exhibited additional neurodegenerative pathology, including p-tau pathology characteristic of ARTAG in one case, and ADNC in another. All 4 individuals with CTE were relatively young at death (in their 50s), and 1 of the 4 died by probable suicide. There was no CTE pathology found in those cases whose brains were referred specifically for dementia research.

**TABLE 3.** Characteristics of CTE Cases Encountered in Routine Practice

	Case 1	Case 2	Case 3	Case 4
Age (decade at death)	Sixth	Sixth	Sixth	Sixth
Sex	Female	Male	Male	Male
Evidence for substance abuse?	No	No	Heavy drinker	No
Evidence for TBI history?	No	History of brain surgery	No	History of boxing
Other conditions associated with TBI?	Epilepsy	No	Epilepsy	No
CTE neuropathology	Frontal lobe –	Temporal lobe –	Frontal, Temporal lobes –	Temporal lobe –
	X 2 lesions	X 1 lesion	X 2 lesions	X 3 lesions
	Low CTE	Low CTE	Low CTE	Low CTE
Other neuropathology	ARTAG; severe CVD; globose tangles in SN and LC; Chaslin’s gliosis	Leptomeningeal fibrosis	No	Hippocampal sclerosis, ILAE type 2; mild white matter gliosis, AD NC (A1 B1 C0)

ADNC, Alzheimer disease neuropathologic change; ARTAG, aging-related tau astrogliopathy; CVD, cerebrovascular disease; ILAE, international league against epilepsy; LC, locus coeruleus; SN, substantia nigra; TBI, traumatic brain injury.

Clear evidence of a prior history of TBI was present in 2 of the 4 CTE cases: one was a former boxer who had sustained multiple head injuries via boxing, and another had a history of TBI via brain surgery and ensuing complications. While the other 2 had no documented evidence of prior TBI in the accompanying notes, they both had a history of treatment-resistant epilepsy with tonic-clonic seizures.

### Sensitivity of 3-Block Restricted CTE Screening

We recently reported on the prevalence of CTE in the first 3 years of the Australian Sports Brain Bank, where 12 of 21 donor brains were diagnosed with CTE (9). However, these diagnoses were made after extensive cortical sampling beyond that employed in our routine autopsy protocol. We thus asked what proportion of these known CTE cases would be detected using only the 3 blocks equivalent to those we used in CTE screening in our routine protocol. In re-examination of these cases using the 3 routine screening blocks only, we found that CTE was detectable in 9/12 (75%) of cases. Of the remaining 3 cases, the “routine” blocks did not contain pathognomonic CTE lesions, although one exhibited sufficient p-tau and other “suggestive features” that would have prompted further investigation (e.g. further block sampling) leading to a CTE diagnosis.

## DISCUSSION

In our standard autopsy neuropathology practice, p-tau IHC was usually only performed when there was a clinical history of dementia or when initial microscopic examination indicated that it may be required and useful. Here we show that incorporating p-tau IHC on 3 routinely sampled neocortical blocks as standard procedure is useful in detecting CTE in general practice. Since establishing p-tau IHC as part of our standard autopsy protocol, we have so far identified 4 cases of CTE from 180 consecutive unselected adult brain autopsies. This equates to roughly a 2% prevalence of CTE in our diagnostic practice.

We cannot say that this figure accurately reflects the prevalence of CTE in the general population of Australia but given the 4 cases found to harbor CTE had a documented or likely history of brain injury, it is consistent with CTE being a disease that is rare outside the setting of prior TBI. In our recent reporting on CTE in donors to the Australian Sports Brain Bank (ASBB) we found a greater than 50% prevalence of CTE in a cohort where all donors had a history of TBI via their involvement in sport (9). Here in our routine autopsy cohort, CTE was found in approximately 8% (2/26) of individuals with a documented history of TBI, and in only 1.3% (2/154) of those without. It is notable that both latter individuals had a long history of treatment-resistant epilepsy with tonic-clonic seizures. Abnormal tau accumulation has been observed previously in autopsy and surgical specimens from individuals with epilepsy (12–14), and while this is not diagnostic of CTE in itself, p-tau accumulation is closely correlated with a history of TBI in epilepsy, and in some instances, bona fide CTE lesions have been observed (15). We were not privy to the full medical history of the individuals reported here (beyond medical notes accompanying the referral) but it is not unreasonable to suppose that the 2 individuals with CTE and no documented history of TBI, did in fact have exposure to TBI as a result of their intractable epilepsy.

It is not possible to apply a meaningful statistical analysis to the relationship between known or likely TBI history and CTE prevalence with only 4 CTE cases in our cohort. However, we were able to detect a 3-fold increase in the diagnosis of any neurodegenerative pathology, including CTE, with a history of TBI. This finding is not unprecedented and is in line with multiple other studies that have linked TBI history to an increased risk of a variety of neurodegenerative conditions (16–20). Although not its primary intent, our report provides yet further evidence linking TBI with an increased risk of neurodegeneration later in life.

Of the 4 CTE cases we report here, only one presented with CTE as the sole neurodegenerative pathology, or “pure” CTE. This individual was 1 of the 2 with epilepsy and also had a history of alcohol abuse. Substance abuse has previously been cited as a factor in CTE and CTE-like pathology encountered in routine practice (21); however, the data from our cohort, which had a high frequency of alcohol and drug abuse history, do not support that concept. Further, while abuse of alcohol or drugs such as opioids may worsen symptomology in CTE (as is the case for many other neurological conditions) there is no evidence that alcohol or opioids cause CTE: p-tau pathology has been previously described in both alcohol abuse (22) and opioid abuse (23), and these pathologies do not resemble CTE.

Diagnostic autopsy for CTE and its characteristic neuropathology has historically been largely restricted to individuals with a history of sports-related concussion or RHI. In this setting, CTE diagnosis involves a very broad sampling of cortical brain regions for p-tau IHC, or even whole hemisphere mounts. The 3-block restricted protocol employed in our routine practice was capable of diagnosing 75% of known CTE cases. This demonstrates that the prevalence of 2% we report here is likely to be an underestimate. However, considering the scant literature on the prevalence of CTE in the general population, the 3-block p-tau CTE screening is a practical trade-off in terms of time and expense for detecting most cases of CTE in routine practice.

The data presented here are consistent with CTE being a disease caused by prior exposure to TBI. Incorporating 3 neocortical block p-tau IHC into routine neuropathology is a cost-effective means to identify CTE in the general population. At the very least, our findings should compel forensic pathologists and neuropathologists to pursue neocortical p-tau IHC in autopsy cases where there is a known or suspected history of prior TBI.

## Notes

There is no funding to report.
